# Effect of Lignin Content on Ultrasound-Induced Nanocellulose Formation in Biorefinery Lignin–Cellulose Mixtures

**DOI:** 10.3390/polym18141697

**Published:** 2026-07-10

**Authors:** Kait Kaarel Puss, Eva-Lotta Palmiste, Nikolai Treiberg, Henry Vider, Siim Pikker, Ilona Faustova, Siim Salmar

**Affiliations:** 1 Institute of Chemistry, University of Tartu, Ravila 14A, 50411 Tartu, Estonia; 2 Institute of Bioengineering, University of Tartu, Nooruse 1, 50411 Tartu, Estonia; 3 Institute of Physics, University of Tartu, W. Ostwaldi 1, 50411 Tartu, Estonia

**Keywords:** wood biorefinery, lignin–cellulose mixture, ultrasound processing, nanocellulose

## Abstract

Lignin–cellulose mixtures (LCMs) generated as intermediates in wood biorefineries are commonly separated into lignin and cellulose. However, using ultrasound (US) to process these mixtures could create novel, valuable materials not possible with conventional methods. This study looked at how lignin affects the US modification of these mixtures. Crude and partially delignified LCMs were successfully prepared using aqueous solutions of EtOH, THF and dilute NaOH and then subjected to short, high-power US treatment. The resulting materials were characterised using FT-IR spectroscopy, particle size analysis, water retention value analysis, SEM and XRD. Sonication rapidly reduced the mean particle size, generating cellulose nanofibril-like structures in all samples according to SEM. The response depended strongly on lignin content, with samples containing lower amounts of lignin exhibiting substantially higher hydration capacity and stronger US responsiveness. At the molecular level, lignin removal exposes cellulose surfaces and enhances hydrophilic interface formation, increasing water uptake and suspension stability. Thus, results show that lignin limits accessible hydrophilic cellulose surface area rather than preventing fragmentation by sonication. US is therefore a chemical-lean strategy to tune the physicochemical properties of partly delignified LCMs and expand the product portfolio of integrated wood biorefineries towards novel advanced lignocellulosic materials.

## 1. Introduction

Lignocellulosic biomass from plants rich in cellulose, hemicellulose and lignin is a valuable source of renewable energy; even more so, it has been considered attractive as a renewable source of chemicals and biomaterials [1,2,3]. The valorisation of lignocellulosic agricultural and forestry residues, or wood processing waste, into biochemicals or smart materials such as nanocellulose [4] or nanolignin [5,6], with novel properties that differ from petrochemistry, has attracted significant interest.

Novel approaches to biorefining, which aim to maximise the conversion of wood biomass into valuable products, have only recently reached an industrial scale [7]. Biorefineries differ from traditional fibre-focused pulp mills mainly in terms of process flexibility, product diversity, and lower environmental impact [2,8,9,10,11]. A variety of pretreatment technologies involving physical and chemical methods can be used by biorefineries [3,8]. One proven option includes steam explosion and weak acid treatment [8,12,13], which break down the wood structure and hydrolyse hemicellulose into monosaccharides. During downstream processing the water-soluble sugars are separated yielding a unique intermediate product lignin–cellulose mixture (LCM), which is the main objective of the current study. LCM has not been used directly as a biorefinery product but has generally been further processed to separate lignin and cellulose components to obtain traditional products. One option is to enzymatically hydrolyse cellulose into glucose for further bioethanol fermentation and so-called hydrolysis lignin (HL) [1,14]. Another approach is to dissolve lignin from LCM with a base and intensively bleach the cellulose fraction into microcrystalline cellulose (MCC) [15].

Recent studies have shown that lignin has properties that improve cellulose materials compared to pure cellulose [16,17,18]. For example, it has been shown that adding lignin to cellulose nanofiber (CNF)-based materials, such as thermoplastics, films, and hydrogels, improves their various properties [19]. Biorefining could allow for flexible and direct control of lignin and cellulose content in LCM [2]. Thus, the direct conversion of LCM into such valuable materials without costly post-processing to separate pure components would expand the biorefinery product portfolio and support the economic viability of the concept.

Ultrasound (US) is a clean and versatile treatment method for process intensification in synthesis, extraction, and the food industry [20,21]. US causes multiple physicochemical phenomena, including heating, acoustic cavitation and streaming, and radical formation. High power US, with a frequency of 20 to 100 kHz, mainly operates through cavitation and streaming, which can disrupt structures linked by hydrophobic or hydrophilic interactions [22,23]. In a lignocellulosic biomass context, US can be applied to increase extraction efficiency [24,25,26], assist in lignin solubilisation [14], or create nanocellulose and nanolignin [5,27,28]. These examples show the versatility of US applicability across processes in the biorefinery.

Previously, we investigated the effects of US treatment on the structure of LCM using a probe system at 20 kHz at varying levels of power, sonication time, and dry matter contents [29]. We demonstrated the formation of nanocellulose structures in LCM without the use of additional chemicals and showed that US-treated LCMs are stable in aqueous media at very high lignin content (>30%). We also found that US had no effect on the depolymerisation or solubility of lignin under the selected ambient and neutral conditions [14,29]. However, the lignin content could play an important role in the energy transfer of US to cellulose crystals by absorbing or scattering the sound and shock waves passing through the solution [27,30,31,32]. To gain further insight into these effects, the lignin content in the LCM can be varied. One way to reduce the amount of lignin in LCM in a controlled manner is to use solvents with different properties. In our recent study, we fractionated HL originating from LCM using mixtures of water-EtOH and water-THF, and characterised the resulting soluble fractions [33]. We found that THF mixtures dissolve lignin more than EtOH mixtures and that the resulting fractions contain molecules with a higher molecular weight. At the same time, HL is most soluble in an alkaline solution and features the largest molecular weight distribution in the soluble fraction [14]. These solvents delignify LCM by targeting specific lignin polymers based on their solubility characteristics.

This study aims to investigate the effect of US on LCM and solvent-treated LCMs with varied lignin content and the properties of these materials. To obtain LCMs with varying lignin contents, lignin was dissolved from wet LCM paste from the biorefinery using selected aqueous solutions of EtOH, THF, and NaOH. The LCM and delignified LCMs were treated with US and characterised by FT-IR, suspension stability, water retention values (WRV), particle size distribution, and particle morphology (SEM, XRD). The effects of lignin content and sonication on LCM properties will be discussed at the molecular level.

## 2. Materials and Methods

### 2.1. Materials

Birch hydrolysis lignin (HL) (lignin content 86.3 ± 1.75%, 1.15% ash, 4.7% glucose, 2.1% xylose, 1.3% galactose, 0.6% arabinose, 1.4% mannose) [14] and birch LCM were provided by OÜ Fibenol (Tallinn, Estonia) and its composition was analysed in this work. LCM paste has 21.7% dry matter and comprises approximately 40.4% lignin, 50.2% cellulose, and 2.5% other wood monosaccharides. Azeotropic EtOH, 99.9% THF, and concentrated H_2_SO_4_ were purchased from Sigma-Aldrich (Burlington, MA, USA). NaOH was bought from Acros Organics (Geel, Belgium).

### 2.2. Delignification of LCM

For delignification, wet LCM paste was weighed to achieve solutions containing 8 wt% of dry matter. The concentrations of the added organic solvents were adjusted according to the water content of the LCM paste to achieve final solvent concentrations of 70% for EtOH and THF and a 0.1 ratio of NaOH (g)/LCM (g). The mixture was stirred for 1 h at ambient temperature (~21 °C).

For organic solvents, after 1 h of mixing, the LCM was centrifuged with an Eppendorf 5804 (Eppendorf, Hamburg, Germany) equipped with a FA-45-6-30 rotor at 4000 rpm (2200 rcf) for 10 min. The solution was decanted, and the residue was washed with the corresponding 70 wt% organic solvent mixture. The mixture was vortexed and centrifuged again with the same conditions for a total of three cycles. Afterwards, the sample was washed three times with water following the same centrifugation conditions.

In the case of alkaline delignification (NaOH), the same centrifugation conditions were applied, and the liquid part was decanted. The insoluble residue was then washed three times with water, and in the last washing step, the pH was adjusted to neutral (pH 7) with dilute H_2_SO_4_.

For both methods, the dry weight of the eLCM (LCM delignified with 70% EtOH), tLCM (LCM delignified with 70% THF), and nLCM (LCM delignified with NaOH) were determined via gravimetry using freeze-drying at −50 °C and 0.3 mbar with an Alpha 1-2 LDplus (Martin Christ Gefriertrocknungsanlagen GmbH, Osterode am Harz, Germany). Dried samples were used for the Klason method and further analysis with FT-IR, SEM, and XRD. The samples without drying were further used for LD and determining WRV.

### 2.3. Determination of Lignin and Cellulose Content

The lignin and cellulose content in all LCM samples was determined by the sulfuric acid hydrolysis method (Klason method). Three individual hydrolyses were conducted, and the Klason lignin content was determined from all three samples. The hydrolysate from one sample set was used to determine the cellulose content directly via HPLC, and in the case of two other sample sets, the cellulose content was calculated from the Klason lignin.

Briefly, 3 mL of 72% H_2_SO_4_ was added to 0.3 g of LCM and mixed for 1 h at 25 °C. Then, 84 mL of ultrapure water was added to dilute the acid solution to 4%, and the mixture was analytically transferred to pressure-tolerant glass reactors to be heated at 120 °C for 1 h. The solution was then cooled to room temperature and filtered with a class filter with a pore size of 16–40 µm using a prefilter with a pore size of 1 µm. The hydrolysate was neutralised with CaCO_3_ and filtered through 0.4 µm into a HPLC vial. A calibration curve was constructed with five common wood monosaccharides: glucose, xylose, galactose, mannose, and arabinose. The instrumental setup consisted of a Shimadzu MS-3030 HPLC, a Shimadzu RID-20 detector (Shimadzu Corporation, Kyoto, Japan), and a Biorad Aminex HPX-87P column (Bio-Rad Laboratories, Inc., Hercules, CA, USA) with a H^+^/CO_3_^−^ deashing guard column. Isocratic flow conditions at a rate of 0.6 mL/min were used, and the mobile phase was MilliQ water.

### 2.4. Fourier Transform Infrared Spectroscopy (FT-IR)

FT-IR spectra were measured using a Spectrum BXII FT-IR spectrometer (Perkin-Elmer, Waltham, MA, USA). The samples were prepared using the KBr disc method, with a sample concentration of approximately 1%. The sample and KBr were finely ground in an agate mortar, and the resulting mixture was pressed under 10 tonnes of pressure to form a disc. FT-IR spectra were recorded with 16 scans in the range of 4000–600 cm^−1^ at a resolution of 4 cm^−1^.

### 2.5. Sonication of LCMs

Based on dry matter determination, the LCM paste was weighed into vials to achieve a final concentration of 0.5 wt%, of which cellulose was 0.3 wt%. Considering their respective composition, the solvent-treated materials (eLCM, tLCM, and nLCM) were weighed into vials to achieve 0.3 wt% of cellulose. The vials were then filled with deionised water to a final volume of 12 mL. The sample was then placed in a cooling jacket, with temperature control at 25 ± 1 °C, and treated with US for 5, 10, or 20 min. The irradiation was carried out with mixing and at 20% of nominal power by a Sonopuls HD 4200 (20 kHz; the maximum power of the US transmitter was 200 W) using the TS103 titanium probe (Bandelin, Berlin, Germany) submerged 1 cm below the level of the heterogeneous mixture.

### 2.6. Particle Size Distribution (PSD)

The non-US and US-treated LCM samples were withdrawn with pipettes as suspensions and directly measured without drying. Particle size distribution (PSD) was determined using a Bluewave S3500 Laser Diffraction analyser (Microtrac, Haan, Germany) with water dispersion at 25 °C. A few drops of sample solutions were put in the machine until the required measurement concentration was achieved. PSD is calculated based on a model that expects particles to be irregular, using the fluid refractive index of water (1.333) and a particle refractive index of 1.

### 2.7. Stability of LCMs Suspensions and Water Retention Value (WRV)

The untreated and sonicated LCM samples were left to stand for 72 h after mixing. The samples were visually assessed for stability, including any signs of sedimentation or phase separation.

The water retention value (WRV) of the samples was measured gravimetrically. First, the samples were centrifuged at 4000 rpm (2200 rcf) for 15 min three times, and the liquid was decanted. The weight of the vials with wet residue was recorded and then freeze-dried at −50 °C under a vacuum of 0.3 mbar until the weight remained constant. The WRV is calculated as grams of water retained by one gram of cellulose and expressed as g_water_/g_cellulose_.

### 2.8. Scanning Electron Microscopy (SEM)

Dried LCM, eLCM, tLCM, and nLCM samples, both non-US and US-treated, were visualised using scanning electron microscopy (SEM). The samples were coated with 7 nm of gold using a SC7640 Auto/Manual High Resolution sputter coater (Quorum Technologies Ltd, East Sussex, England) using 1.5 kV 15 mA for 90 s. Imaging was performed with a Nova NanoSEM 450 (Thermo Fisher Scientific Inc., Waltham, MA, USA) equipped with a CBS detector. Measurements were made with an electron beam energy of 5 kV, with a dwell time of 10 µs.

### 2.9. X-Ray Diffraction (XRD)

Dry samples were prepared on top of a silicon wafer with a sample thickness of 1.5 mm. Measurements were made using a D8 Advance instrument with Cu-Kα radiation, 2.5θ Soller pulses, equipped with LynxEye detector and data were analysed with DIFFRAC.EVA V7 and Diffrac.Suite TOPAS 6, 2017 (Bruker Corporation, Billerica, MA, USA). The measured range was 5–60° 2θ with a step of 0.018° 2θ and a summative time of 60 s per datapoint. Cellulose standard used (Cellulose Iβ 00-060-1502) is from the ICDD database PDF-5+ (2026).

## 3. Results and Discussion

### 3.1. Delignification and Characterisation of LCMs

First, LCM should not be confused with LCC (lignin–carbohydrate complex), which refers to a covalently linked structural complex between lignin and carbohydrates (mainly hemicelluloses and sometimes cellulose) [34]. Within the biorefinery process, wood biomass is converted via thermo-chemical-mechanical and acid hydrolysis treatment into a mixture of lignin and cellulose, wherein hemicellulose is converted into monosaccharides. In the LCM used in this work, less than 2.5% of hemicellulose residual sugars were determined as xylose, the main monosaccharide of birch hemicellulose [35]. Nevertheless, the subsequent procedure of lignin dissolution leads to the complete elimination of free hemicellulose sugars from the solvent-treated LCMs. Thus, this study does not focus on the covalent molecular complexes between lignin and carbohydrates.

To examine the effect of lignin content on the sonication of crystalline cellulose in LCM, different solvents were used for delignification. Based on our previous studies on the dissolution of HL, we selected 70 wt% water-EtOH, 70 wt% water-THF [33], and 0.8% NaOH [14] solutions. While both 70 wt% EtOH and THF showed maximal HL dissolution at this concentration, THF was found to be more effective at dissolving lignin and extracting higher molecular weight lignin. Structural analysis (2D-HSQC NMR) revealed similar lignin subunits and bond types in the two organic solvent fractions. However, ^31^P NMR analysis showed that the EtOH-soluble lignin had a higher OH content and polarity. Consequently, residual lignin in LCM may be more hydrophobic after EtOH treatment than after THF treatment. Aqueous NaOH solution is a known effective lignin solvent and allows for the extensive delignification of LCM.

Compositions of non-treated and solvent-delignified LCMs are presented in Table 1. Similarly to the HL dissolution in our previous work [14,29,33], the decrease in lignin from LCM is lower with 70 wt% EtOH than 70 wt% THF, which increases the cellulose content in the corresponding insoluble fractions, eLCM and tLCM. The aqueous NaOH treatment decreases the lignin content the most, and the resulting LCM material (nLCM) has only 15.2% lignin left. Moreover, the cellulose content is the highest in nLCM, reaching 75.3%. These analyses confirm that solvent treatment is an effective method to dissolve lignin selectively from LCM, while the cellulose properties are expected to remain largely unchanged [36]. The prepared LCMs were used to investigate how sonication affects their structure and properties, with lignin content being one of the variable factors. The results are discussed in the following sections.

**Figure 1:**
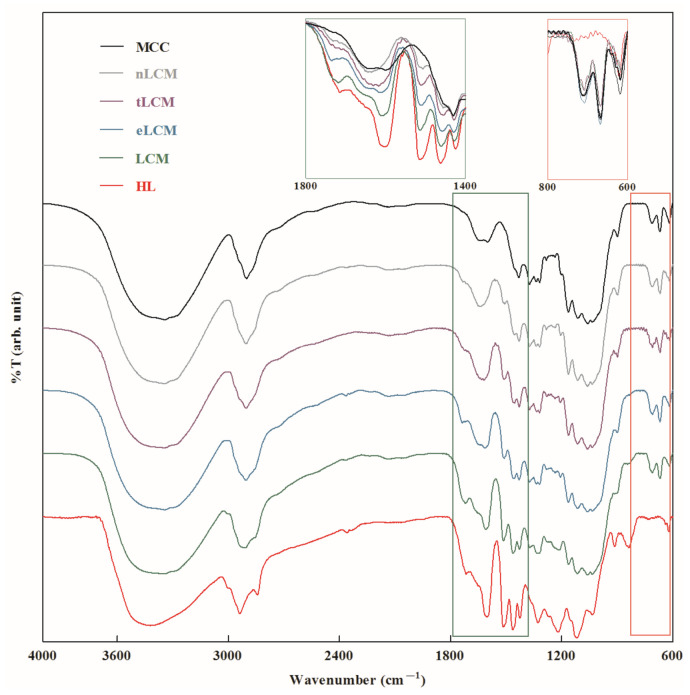
FT-IR spectra of isolated MCC, HL, LCM, eLCM, nLCM, and tLCM. The peaks that are characteristic of lignin (1800–1400 cm^−1^) and crystalline cellulose (800–600 cm^−1^) are highlighted in inserts, with the corresponding spectral parts presented as overlapping.

In addition to the Klason method for composition determination, FT-IR spectroscopy was employed to analyse the structural differences between the studied LCMs. This analysis provides additional insights into the lignin content and other structural changes that may occur in LCMs during delignification. A HL sample and an MCC sample are included in Figure 1 for comparison, which allows the identification of lignin-specific and cellulose-specific signals in the FT-IR spectra of the LCMs.

Based on the spectra and literature, the main lignin-specific signals are in the range of 1700, 1600, and 1210 cm^−1^. The broad peak from 3800 to 2400 cm^−1^ corresponds to the O-H stretching vibration, which is present in both lignin (in the case of HL, more phenolic OH groups shift the band to the left or higher wavenumbers) and cellulose (only aliphatic OH groups). The signal at 2935 cm^−1^ is attributed to C-H stretching. The 1700 cm^−1^ signal corresponds to C=O stretching, while the 1600 and 1515 cm^−1^ signals are due to aromatic C=C skeletal vibrations, which are indicative of lignin content reduction in LCM. However, the adsorption peaks of lignin and cellulose overlap in many respects in this area, which may make it difficult to draw uniform conclusions. There is a less intense peak of cellulose at around 1640 cm^−1^, which apparently causes the adsorption band to shift from wavenumber 1600 cm^−1^ to 1640 cm^−1^ as the amount of lignin in LCM decreases. The band at around 1220 cm^−1^ corresponds to lignin C-C and C-O stretching vibrations [37,38].

The main cellulose peak at 1060 cm^−1^ (C-O and C-O-C stretching) appears consistently across all LCM materials and is less prominent in the HL sample. Similar linkages are also present in HL, but the lower signal intensity is consistent with previous observations. The signal at 897 cm^−1^ corresponds to the amorphous region of cellulose [39]. Additionally, the signals at 1370 cm^−1^ and between 800 and 650 cm^−1^ correspond to the crystallinity of cellulose [40]. However, calculations based on dynamic FT-IR were not performed here, so the ratio of signals was not evaluated. We observed a relative increase in the 1370 cm^−1^ signal to the decreasing lignin content, with the change being more pronounced in the tLCM and nLCM samples compared to the LCM and eLCM. The bands at 670 cm^−1^ and 710 cm^−1^, where the HL spectrum shows no absorbance, had same intensity for all LCM samples, which implies that solvent treatment did not change the structure of cellulose after lignin separation.

### 3.2. Effect of Ultrasound Treatment on LCMs

#### 3.2.1. Sonication and Characterisation of LCMs

In recent work, we found that the practical application of US treatment and its effects are greatly affected by the dry matter content of LCM in the aqueous mixture (0.5–4 wt%; the ratio of lignin to cellulose was constant) [29]. A higher LCM content caused the mixture to gel upon sonication, increasing its viscosity and inhibiting the transfer of US energy. The dry matter content also directly affects the morphology of sonicated LCM, resulting in the formation of nanocellulose structures and a change in the water retention value (WRV; see below). However, the impact of lignin content on US treatment and the properties of the resulting materials requires a more profound understanding. To maximise the sonication effect on cellulose, a low concentration of 0.5 wt% LCM in water was chosen as the reference in this work. Thus, the lignin to cellulose ratio in LCM is the same as that produced in a biorefinery (approximately 0.7–0.8). In eLCM, tLCM, and nLCM, however, this ratio is lower due to delignification. Before sonication, all samples were prepared so that the cellulose content in the suspension mixture was 0.3 wt% based on the composition obtained by the Klason method (Table 1). This standardisation facilitates comparison across LCMs and ensures that any observed effects can be attributed to lignin content variations.

**Figure 2:**
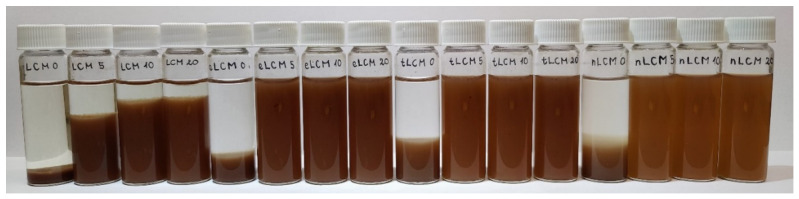
The stability of the non-sonicated (0 min) and sonicated (5, 10, 20 min) LCM, eLCM, tLCM and nLCM suspensions after 72 h from their preparation.

Figure 2 shows the LCM suspensions, both mechanically stirred and sonicated for 5, 10, and 20 min, which were then left to stand for 72 h at ambient temperature to check their suspension stability. Cellulose is intrinsically hydrophilic and interacts strongly with water, resulting in the high water absorption capacity of cellulose-based materials. This capacity depends on the water-accessible surface area of cellulose, which can be significantly increased through mechanical fibrillation or sonication, thereby enhancing water uptake. It is well established that the nano- and micro-fibrillation of cellulosic fibres results in colloidally stable suspensions or gel formation. Thus, the stability of cellulose suspensions may be employed to assess the degree of fibrillation [41,42]. The colour of the samples is affected as the lignin content decreases from LCM to nLCM, resulting in lighter brown hues. Evidently, a brief sonication enhances the stability of the resulting dispersions, and in the case of LCM treatment, dependence on irradiation time is also observable. With 5 min of sonication, the LCM suspension is less stable than with 10 min, and with further sonication up to 20 min, it stays the same or is marginally better.

Interestingly, the samples without sonication clearly exhibit different dispersion stability depending on the lignin content. While the LCM without solvent treatment has precipitated, forming the densest sediment layer, the sediment level of the ethanol-treated eLCM is twice as high. The sediment level is even higher in the case of the tLCM and nLCM. It could be concluded that reducing hydrophobic lignin content with solvents and thus ‘washing’ the cellulose crystals releases hydrophilic surfaces or areas of polymers that readily interact with the structure of the water to form more stable dispersions [43]. Moreover, the reduced lignin levels in eLCM, tLCM, and nLCM enhance the stability of the dispersions, as no apparent precipitation is observed in these sonicated samples. Despite the expectation of differing settling rates for lignin and cellulose particles, distinguishing between them was not possible. Nevertheless, the homogeneity of the original material may also be sustained during the settling process, thus particles consist of both biopolymers. At the advanced or nano-fibrillation stages, the increased fibril surface area enhances interactions with water, reducing the sensitivity of suspension stability as a measure of fibrillation degree [42], which seems to be the case for the US-treated samples as well.

No significant chemical changes were revealed in the US-treated samples by IR spectroscopic analysis compared to those without sonication, regardless of the solvent used for delignification, as the FT-IR spectra were similar (Figure S1). Additionally, no significant changes were observed in the 670 cm^−1^ peak, which is characteristic of crystalline cellulose. Therefore, it is possible that sonochemical oxidative impacts are not superior within such a brief sonication timeframe in comparison with mechanical ones.

#### 3.2.2. Water Retention Value of Treated LCMs

**Figure 3:**
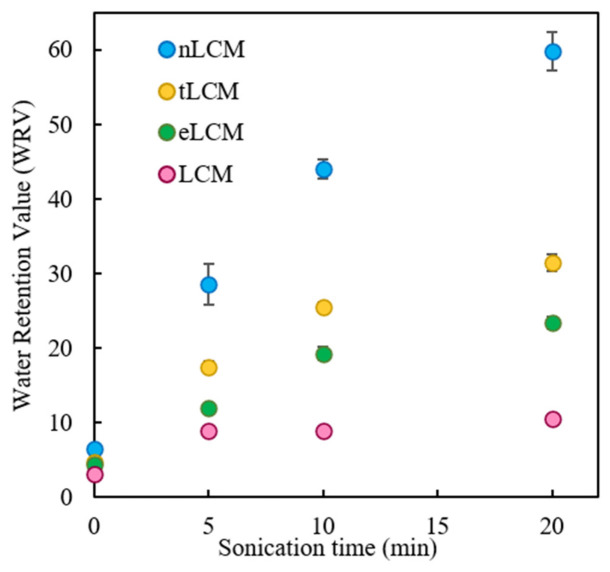
WRV (g_water_/g_cellulose_) for LCM, eLCM, tLCM and nLCM with equalised cellulose contents plotted against the sonication time. Numerical values with errors are presented in Table S1.

The water retention value (WRV) reflects the ability of fibres to retain water and is widely used in the pulp and paper industry to assess fibre fibrillation. WRV increases with refining due to enhanced fibre swelling from internal fibrillation and delamination, as well as increased external surface area from external fibrillation. Consequently, WRV is commonly applied as a semiquantitative indicator of the degree of micro- and nano-fibrillation and the overall surface area of cellulose samples [42,44]. In the case of LCM, WRV provides useful info, e.g., on the capacity to hold water via hydrogen bonds in cellulose and how this capacity is altered by changes in lignin content or by sonication. Figure 3 shows the variation in WRV of LCM materials as a function of US irradiation time.

Without sonication, the WRV increases with decreasing lignin content from LCM to nLCM, more than twice from 3.0 to 6.4. Although the change in VRW is relatively small (Figure 3), the trend correlates with the results of the LCM suspension stability in Figure 2. This directly hints at the effect of lignin content on WRV. However, the WRVs are very close for eLCM and tLCM, at 4.2 and 4.4, respectively. This may also indicate the separation of lignin with a different hydrophobicity when treated with these two solvents.

However, even at very short sonication times, the WRV increases significantly for both LCM and solvent-treated LCMs. For the LCM, the WRV reaches its maximum after 5 min and remains unchanged upon further sonication. These findings are similar to those achieved in our earlier research, where under different US conditions at various intensity levels, WRV attained a plateau, demonstrating the distinctiveness of LCM [29]. In contrast, for nLCM, WRV is highest after 20 min and is almost 10 times higher than that of the untreated LCM sample (6.4 and 59.7, respectively). A similar continuous increase in WRV with respect to sonication time is shown by all other solvent-delignified LCMs, but a near plateau is also approached after 20 min. The increase in the WRV is in the same direction as the depth of delignification. These results indicate that lignin plays a crucial role in how physicochemical effects alter cellulose structure and properties during sonication.

**Figure 4:**
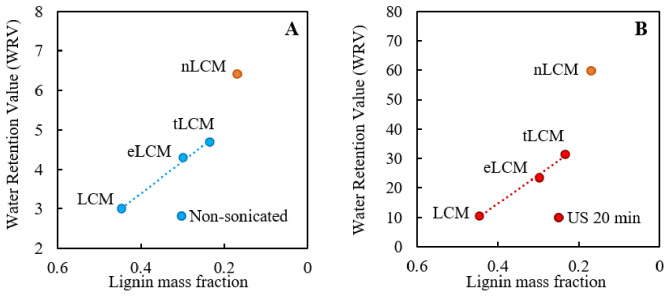
WRV (g_water_/g_cellulose_) of LCM, eLCM, tLCM, and nLCM plotted against lignin mass fraction, which is calculated with equation m_lignin_/m_lignin_ + m_cellulose_. The dependence depicted in panel (**A**) is for non-sonicated LCMs, and in panel (**B**) for LCMs after 20 min of sonication.

To assess the sensitivity of WRV to the lignin content in LCMs, lignin concentrations, expressed as lignin mass fraction (m_lignin_/(m_lignin_ + m_cellulose_) in the corresponding LCMs, were plotted against their WRVs (Figure 4).

Figure 4A shows the effect of lignin content on WRV of LCMs without sonication. While the increase in WRV is well correlated with the decrease in lignin content in the case of LCM, eLCM, and tLCM, the nLCM shows a significantly higher WRV. This can be explained by the nature of WRV, which is related to water interactions. Inert EtOH and THF remove hydrophobic lignin from LCM without directly affecting the cellulose or residual lignin chemically or physically. Severe swelling (mercerization) of the fibres can be caused by the use of high concentrations of NaOH, which breaks intermolecular hydrogen bonds [45]. This structural loosening exposes more hydroxyl groups and improves water uptake. However, such an effect is not expected with 1% NaOH used in this work. It has also been suggested that WRV is affected by lignin removal methods in the case of lignin-containing cellulose by increasing the hydrophilicity of the residual lignin [42]. Figure 4B shows a similar relationship between WRV and lignin mass fraction after 20 min sonication, albeit with a higher effect of US when there is less lignin. Consequently, the WRV of LCM increases by 3.5 times, but the WRV of nLCM increases even 9.3 times higher than their non-sonicated counterparts. This indicates that both the overall lignin content and the nature of lignin play a role in the US effect on WRV of LCMs. This demonstrates that while WRV may not be the optimal approach for precisely gauging the US impact on cellulose in LCM, it still offers insights into how to tailor the properties of LCM materials to meet specific applications.

#### 3.2.3. Particle Size Analysis of LCMs

The change in particle size distribution (PSD) can provide additional information about the effect of US on LCM at different lignin contents, which can be measured using laser diffraction (LD) method. This is a powerful indicator of how pure nanocellulose (e.g., CNF or CNC) [46,47] or nanolignin [48] evolves during its preparation with sonication and also shows manufacturing efficiency. Unlike electron microscopy (SEM or TEM), which requires extensive sample preparation, including drying the samples, the LD method can be used directly on the dilute suspension. LD is usually not well-suited for determining a true morphology-based PSD in heterogeneous suspensions like LCM, consisting of particles from wood cell structure, cellulose crystals, particles composed of lignin polymers, or associated particles of both biopolymers. LD cannot distinguish between these populations and treats aggregates as single particles, and optical property ambiguity introduces systematic errors [49]. Although the absolute PSD of LCM and solvent-treated LCMs cannot be determined by LD, it could be useful for relative comparisons and trend analysis of the US effect on the LCM samples with the same composition.

**Figure 5:**
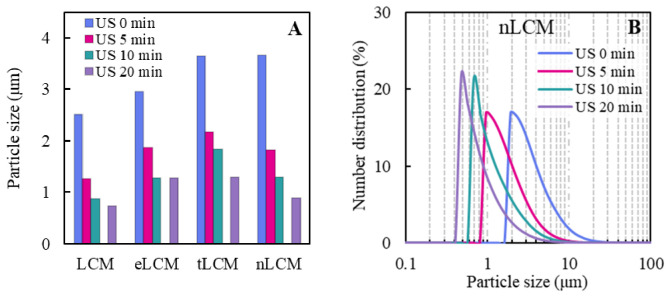
(**A**) The number-weighted average particle sizes of LCM, eLCM, tLCM, and nLCM without (0 min) and after 5, 10, and 20 min of sonication. (**B**) Number-weighed particle size distribution of nLCM without (0 min) and after 5, 10, and 20 min of sonication. Percentiles of distributions are presented in Table S3.

For all LCMs, the LD results before and after sonication showed a significant exponential-like reduction in mean particle size (Figure 5A). The corresponding numerical data are tabulated in Tables S2–S5.

Figure 5A shows that sonication reduced the mean particle size by up to two times after just 5 min. Further sonication for up to 10 min results in a continued reduction in particle size, though to a lesser extent than after the initial 5 min. The effect of continued sonication for up to 20 min on the particle size is more moderate, or has no effect at all, indicating that an optimal or minimal particle size is very close to being achieved under the sonication conditions used. However, it is suggested that long sonication times can cause extensive oxidation, generating radicals that can initiate polycondensation of lignin through a radical process [50]. The sonication process can lead to the formation of phenoxy radicals by phenolic hydroxyl (OH) groups in lignin, which can trigger cross-linking reactions [51]. It has also been shown that cellulose is converted into water-soluble compounds upon sonication over a long period of time [52]. Therefore, to avoid the possibility of the lignin radical-induced polymerisation and the formation of cellulose oligomers, shorter sonication times were used in this study.

It is clearly demonstrated by the LD results that regardless of the nature and composition of large particles or aggregates in LCM suspensions, the energy generated by cavitation is more than sufficient for them to be broken down into small particles. It is important to emphasise that a comparable pattern of alteration in particle size was evident in all LCMs, irrespective of the lignin content, as exemplified with the nLCM in Figure 5B and in Supplementary Information Figures S2–S4. Furthermore, the size distribution appears to be associated with the stability of suspensions (Figure 2) and the WRVs (Figure 3). The growth in the number of smaller particles increases the stability of the suspensions, and the WRV increases accordingly with sonication over time. Although the LD method has its limitations, it still reveals certain patterns. For the LCMs without sonication, the particle sizes increase in the direction from LCM to nLCM (see Figure 5A), thus corresponding with the decrease in lignin content in LCMs. This suggests that lignin molecules form smaller hydrophobic aggregates with each other or with cellulose particles, which are detectable by the LD method, but whose dissolution shifts the average particle size of solvent-treated LCMs to a larger value (compare Figure 2 and Figure 5A). Even though the mean particle size of non-sonicated LCMs rises with a reduction in lignin content (Figure 5A), the stability of the suspension is enhanced, and sedimentation is mitigated (Figure 2). This means that lignin affects how cellulose and water interact, thus lignin removal leads to the better water accessibility of cellulose. Although US significantly reduces the particle size of crude LCM, with the maximum effect seen after a short irradiation time, the WRV does not change with further irradiation. The change in particle size caused by sonication is directly related to the increase in the stability of LCMs water suspensions and somehow the increase in their WRV. However, the LD method alone is not enough to account for the impact of lignin content in LCM on the US effect and the resulting changes in WRV. More clarification can be provided by the morphological analysis of US-treated materials using the SEM method.

#### 3.2.4. Morphologies of LCMs Characterised by SEM Analysis

**Figure 6:**
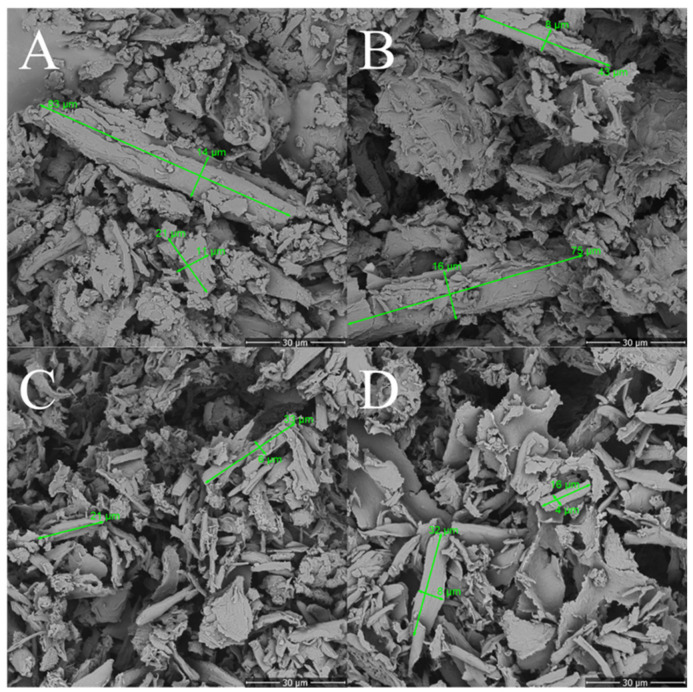
SEM micrographs of LCM (**A**) and delignified eLCM (**B**), tLCM (**C**), and nLCM (**D**) at 2000× magnification.

The morphological changes in LCM subjected to progressive lignin removal were investigated using SEM analysis. As SEM analysis requires samples with low moisture content; all samples were thoroughly dried before analysis. However, it is noteworthy that drying could lead to cellulose hornification [24], which may affect the observed morphological characteristics. Nevertheless, SEM micrographs expose significant differences between the untreated and solvent-treated LCMs seen in Figure 6.

All non-sonicated samples appear to be heterogeneous, containing elongated structures of varying sizes, and a crusty, sticky material is seen on the surface of these particles and in clusters between them. This suggests that lignin covers the cellulose structures, resulting in more of the rougher structures. The initial LCM (Figure 6A) material consists of micro-sized particles heavily coated with a crumbly texture and an amorphous mass on the surface. The cellulose or wooden structural particles seem to be masked by the lignin. The micrographs (Figure 6A,B) also show very large wood cell wall particles, which are common in biorefining industry slurries using steam explosion technology or similar. Upon the partial removal of lignin with EtOH (Figure 6B), the microstructure begins to show signs of delamination. Some microstructure features become distinguishable, indicating the onset of lignin dissolution. At higher lignin removal with THF (Figure 6C), the surface of particles becomes increasingly smoother, with significantly more exposed, sharper elements. The expected cellulose microcrystals become more apparent with sharper edges and increased separation between individual components. The reduction in agglomeration suggests a substantial decrease in lignin adhesive-like influence. Removing lignin with NaOH (Figure 6D), corresponding to near-complete delignification, the image reveals a highly regular and layered structure. The particles appear sharp-edged and well-defined, with minimal amorphous-type coating. The visual change is consistent with the expected behaviour of LCM after delignification and supports the FT-IR analysis based on the change in chemical composition. The delignification step presumably cleans the hydrophilic surfaces of cellulose, which could promote interactions with water and result in an increase in suspension stability and WRVs (Figure 2 and Figure 3).

**Figure 7:**
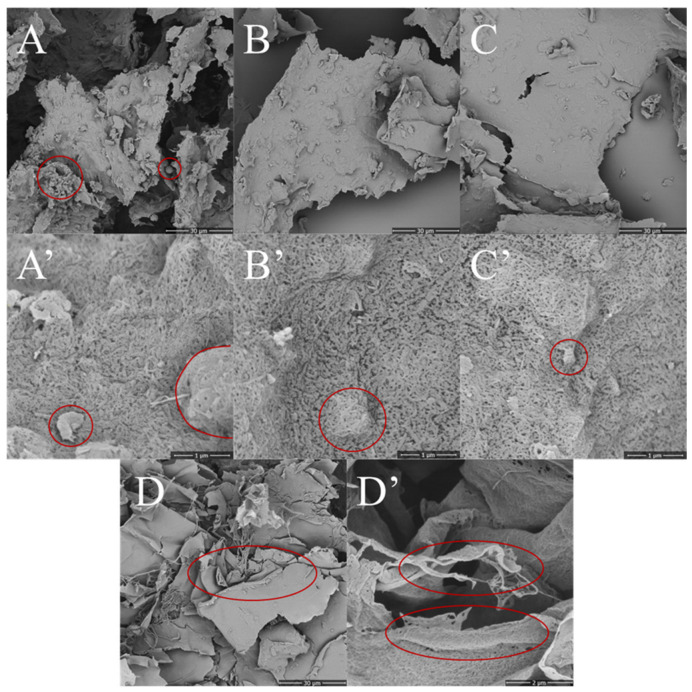
SEM micrographs of LCM (**A**), eLCM (**B**), tLCM (**C**) and nLCM (**D**) after 20 min of sonification. The micrographs (**A**–**D**) are at 2000×, (**A’**–**C’**) at 50,000×, and (**D’**) at 30,000× magnification. Red circles indicate some examples of key morphological findings.

As Figure 7 shows, sonication breaks down the macroscopic structure in all cases, which is consistent with previous results [53]. Large fragments or structural pieces of wood cells (Figure 6) are no longer visible. The dimensions of the more homogeneous laminar particles are mainly related to the drying method and not to the direct result of sonication (Figure 7). The higher magnification micrographs (A’, B’, C’, D’) in Figure 7 provide a closer look at these structures, revealing that the sheet-like formations are composed of layers of fibrils arranged on top of each other. The sheets are bridged by individual fibres, where measurements can be taken to confirm the presence of CNF. Nevertheless, the dimensions of the CNFs forming the layers appear similar for all LCM samples. However, a difference in structure resulting from solvent delignification is clearly visible. Although the sonicated LCM (Figure 7A,A’) no longer contains elongated particles with sharp edges, they are still less homogeneous and contain jagged bumps and rounded formations, which can be linked to the higher lignin content. It is clearly seen that with eLCM (Figure 7B,B’) and tLCM (Figure 7C,C’), the uneven bumps disappear and the CNF structures appear in the observed thin layers. However, in the case of nLCM (Figure 7D,D’), a certain difference can be observed. Although the presence of identical sheets of cellulose is seen, as in all other samples, pure, clearly separated nanoscale bundles and fibrils consisting of smaller CNFs also appear. Thus, the exposure of extra cellulose fibre surface is a result of delignification and sonication, which may provide an extra explanation for the higher WRV in the case of nLCM (Figure 4B). In conclusion, US causes microparticle defibrillation in all LCMs to CNF regardless of lignin content, which is expected to result in an increased hydrophobic surface area of cellulose. This promotes the interaction of these materials with water, resulting in more stable suspensions and an increased WRV.

#### 3.2.5. Crystal Structure Analysis of LCMs

**Figure 8:**
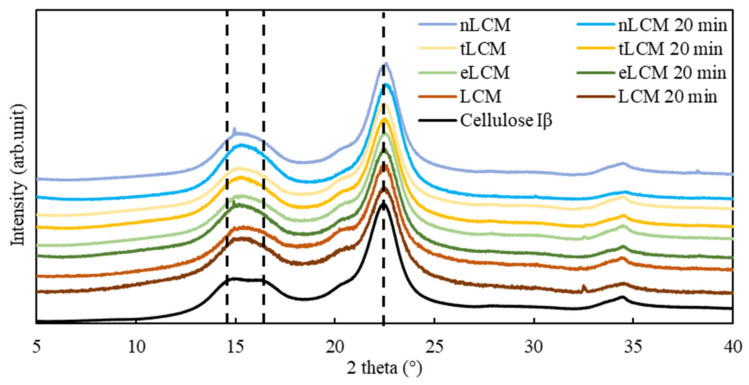
X-ray diffraction patterns of LCM, eLCM, tLCM, and nLCM without and after 20 min of sonication. The cellulose Iβ diffractogram (black line) is from International Standards (ICDD database PDF-5+, 2026) and is for comparison. Vertical dashed lines highlight the diffraction peaks for cellulose Iβ.

The crystalline behaviour of the dried LCMs non-sonicated and after 20 min of sonication was analysed with XRD, which enables us to determine the type of cellulose present in the sample by matching the sample diffraction patterns with the standard from the database (ICDD). In the case of LCMs, the measured diffraction patterns were found to match most closely the cellulose Iβ diffraction pattern (Figure 8). The characteristic diffraction peaks of cellulose Iβ at 2θ are around 14.5°, 16.5°, and 22.5° [41,54,55], and it can be seen from the diffractograms that neither solvent delignification nor sonication changes the crystal structure. XRD analysis also indicates no swelling of the cellulose fibres due to the NaOH treatment, which would alter the Iβ structure. Therefore, this is most likely not the reason for the increase in the WRV in case of nLCM. However, the small shift in the samples compared to the theoretical diffraction pattern is likely due to the low density of the LCM samples. X-rays can penetrate softer materials deeper, resulting in wider reflection peaks. The roundedness of the peak around 15° 2θ is due to the amorphous nature of the lignin, which reduces the signal-to-noise ratio.

**Figure 9:**
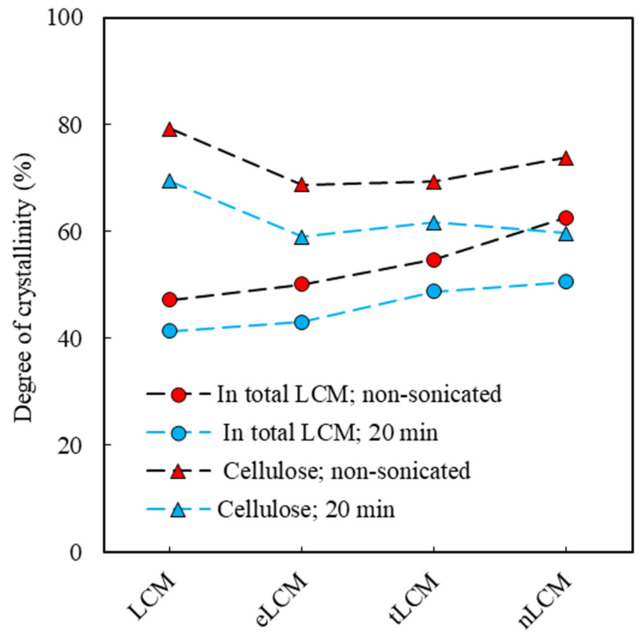
XRD-measurement-based empirical calculations of degree of crystallinity using cellulose Iβ (00-060-1502 from ICDD database). Crystallinity in the total LCM materials at different lignin contents without and after 20 min of sonication (marked by circles); crystallinity of cellulose where the amorphous lignin content has been subtracted (marked by triangles) without and after 20 min of sonication.

As lignin is fully amorphous, and a part of cellulose is likewise amorphous, the total diffractogram comprises the intensity of crystalline cellulose, amorphous cellulose, and lignin. The degree of crystallinity can be obtained by fitting the diffractogram of cellulose Iβ as a standard using curve-fitting software (Bruker Diffrac.Suite TOPAS 6) to separate these contributions [45,56]. If amorphous cellulose and lignin are considered as the same amorphous fraction of the material, then the crystallinity corresponds to the fraction of crystalline cellulose in the total LCM sample. As the lignin fraction is known from chemical analysis (Table 1), the corrected crystallinity of cellulose can be calculated by subtracting the known lignin fraction from the total amorphous fraction. The corresponding results are presented in Figure 9.

As can be seen on Figure 9, the effect of solvent treatment on the crystallinity of cellulose is largely negligible. Surprisingly, with nLCM, a minor increase in crystallinity can be seen, which had a similar sharp increase in WRV (Figure 3) compared to eLCM and tLCM. Most likely these effects could be attributed to the removal of critical amount of amorphous lignin, which significantly increases hydrophilic surface area of cellulose. However, while unlikely, structural alterations brought upon by alkaline-induced swelling cannot be completely ruled out either. Nevertheless, in all cases, sonication decreases crystallinity, which in turn increases the amount of amorphous cellulose, the part responsible for water retention.

In native cellulose, amorphous regions are believed to be present both on the surfaces of the elementary fibrils and in the areas between crystalline domains. NMR studies suggest that the cellulose chains at the crystallite surfaces differ structurally from those in the core, and molecular dynamics simulations indicate that these surface molecules are less ordered than those in the interior [45]. Thus, US can also increase the amorphous phase by creating new hydrophilic surfaces when breaking longitudinal fibres.

#### 3.2.6. Molecular Considerations of the Effects of US Treatment and Lignin Content on LCMs

The structure and crystallinity of cellulose fibres have been previously characterised using techniques such as XRD, NMR spectroscopy, and other related analytical methods [45,57,58,59]. These studies provide detailed descriptions of the molecular organisation of cellulose. Molecular dynamics simulations have further examined the interactions between cellulose fibres and lignin molecules, which contribute to understanding how lignin associates with cellulose surfaces and influences intermolecular interactions within lignocellulosic materials [60,61]. Together, these findings enable consideration of the mechanisms underlying US effects on LCMs, and explain how variations in lignin content influence material properties at the molecular level.

It has been established that the molecular structure of cellulose Iβ crystals is primarily defined by the arrangement of the unit cell and the corresponding crystal planes [45]. The proposed structure of the elementary fibril of cellulose Iβ is hypothesised to be hexagonal, with the outer surfaces of the fibril categorised as hydrophobic or hydrophilic. The (100) and (200) faces are considered hydrophobic in nature, as these surfaces cannot form hydrogen bonds due to the orientation of the hydroxyl groups. As the rest of the designated fibril faces, (110), (1–10), and (010), are more than twice as large and can form hydrogen bonds abundantly, they are considered hydrophilic to a varying degree [62]. Microfibrils are constituted by elementary fibrils, thus the interactions with water and hydrophobic lignin are determined by the characteristics of the corresponding planar surfaces [63].

It is proposed that during sonication, cellulose microcrystalline particles are scissored crosswise in the amorphous region into shorter fibres; even more so, they are broken into fine CNFs along the fibre direction [64,65]. The molecular structure of cellulose fibril indicates a correlation between the substantial WRV increase caused by sonication and the formation of new hydrophilic surfaces to a very large extent. SEM analysis demonstrates the formation of CNF with highly comparable dimensions for all LCM samples following 20 min of sonication (Figure 7). However, the WRV growth experiences a decline until it reaches a maximal state during sonication at higher lignin contents (Figure 3).

Recent computer simulations of lignin–cellulose interactions in water show that the adsorption of lignin is energetically favoured mostly on the hydrophobic face of cellulose, but due to the amphiphilic nature of lignin, interactions between the hydrophilic face and lignin are still possible [60,61]. These observed molecular considerations may elucidate why sonication has less of an effect on the WRV of LCM, which has the highest relative lignin content (Figure 3). It can be argued that the hydrophilic parts of the lignin polymer compete with water for the hydrophilic surfaces of the cellulose crystals. At high lignin levels, a hydrophobic lignin layer can form on the surface of the cellulose fibres, and access to water is limited, resulting in a lower WRV. Lignin removal from eLCM and tLCM, however, frees the hydrophilic surfaces of the cellulose, allowing for more interactions with water, and WRV increases linearly with decreasing lignin content (Figure 4). In the case of nLCM, a significant increase in WRV occurs even without sonication. This may be related to the partial swelling of the fibres by NaOH, which could also free the hydrophilic surfaces of fibres [45], or there is simply too little lignin to compete with water.

## 4. Conclusions

This study demonstrates that LCM, an intermediate produced in an industrial wood biorefinery, can be converted into stable, nanostructured, cellulose-rich suspensions and hydrogels through short sonication, without the need for additional reactive chemicals.

The delignification of the LCMs using aqueous EtOH, THF, and dilute NaOH resulted in a systematic reduction in lignin contents (from ~40% in LCM to ~15% in nLCM), while maintaining the integrity of the cellulose Iβ crystal type. FT-IR analysis confirmed the progressive removal of lignin, while cellulose-associated bands remained consistent. This suggests that solvent washing primarily altered the composition of the material, rather than fundamentally changing the chemistry of cellulose.

Sonication rapidly and effectively modified LCMs by fragmenting the heterogeneous particles, producing a pronounced reduction in average particle size within the first 5–10 min, improving suspension stability, and promoting fibrillation of the cellulose-rich fractions. WRV increased with decreasing lignin content even without sonication, indicating that lignin removal improves the water accessibility of cellulose. Sonication further amplified WRV for all LCM suspensions, but the response depended strongly on lignin content. SEM analysis corroborated these trends by revealing the extensive breakdown of microscopic wood fragments and the formation of layered CNF-like sheets in all sonicated samples, while nLCM uniquely showed more clearly separated nanoscale fibril bundles, which is consistent with the highest WRV and strongest hydration capacity. XRD analysis indicated that a modest decrease in apparent crystallinity after sonication was observed, particularly for nLCM, which is consistent with cavitation-driven scission and the generation of new, less-ordered surface regions as fibrils are liberated. Taken together, the results support a mechanism in which sonication effects create new cellulose surface area, but high lignin content limits the accessible hydrophilic surface by preferential lignin adsorption and shielding effects. The results indicate that lignin does not prevent US-induced fragmentation but reduces the attainable WRV and diminishes the incremental benefit of longer sonication. Partial delignification enhanced the response of LCM to sonication; whereas, the most extensively delignified material showed the highest water retention and the strongest overall structural response. This supports the interpretation that lignin acts as a surface-associated barrier that moderates cellulose–water interactions under sonication.

Overall, the content of lignin is a key parameter that governs the physicochemical outcome of scalable US processing of LCM. These findings demonstrate for the first time that US can be used to tailor the properties of the biorefinery intermediate LCM, upgrading it directly into innovative functional lignin-nanocellulose materials without requiring complete fractionation into pure components. The sustainability of integrated biorefinery concepts is increased by this approach through the diversification of the product with valuable materials such as hydrogels, aerogels, sorbents and filters that could find applications in cosmetics, medicine and other fields.

## Figures and Tables

**Table 1:** Composition of the non-treated LCM and solvent-treated LCMs. The values of Klason lignin and cellulose contents are presented in the table and expressed on a dry mass basis with standard deviations.

Material Code	Delignifying Solvent ^a^	Klason Lignin (%)		Total Lignin	Cellulose (%)
		AIL (%)	ASL (%)		
LCM	-	38.3 ± 1.1	2.1 ± 0.3	40.4 ± 1.3	50.2 ± 1.6
eLCM	70 wt% EtOH	26.1 ± 2.4	1.1 ± 0.4	27.1 ± 2.0	63.9 ± 2.6
tLCM	70 wt% THF	20.3 ± 0.6	0.8 ± 0.1	21.1 ± 0.6	69.1 ± 0.6
nLCM	0.8% NaOH	14.6 ± 0.2	0.6 ± 0.2	15.2 ± 0.4	75.3 ± 0.9

^a^ aqueous solution.

## Data Availability

The original contributions presented in this study are included in the article/Supplementary Materials. Further inquiries can be directed to the corresponding author.

## Supplementary Materials

The following supporting information can be downloaded at: https://www.mdpi.com/article/10.3390/polym18141697/s1.
