# Supplementary material for: Effect of Lignin Content on Ultrasound-Induced Nanocellulose Formation in Biorefinery Lignin–Cellulose Mixtures
Source: Polymers (Basel). 2026 Jul 10;18(14):1697. doi: 10.3390/polym18141697 (PMC13431309; doi:10.3390/polym18141697)
Supplement: Supplementary file 1 [file polymers-18-01697-s001.zip › polymers-4319511-supplementary.pdf]

Supplementary data

Effect of Lignin Content on Ultrasound-Induced Nanocellulose Formation in Biorefinery Lignin–Cellulose Mixtures

Kait Kaarel Puss [1,2], Eva-Lotta Palmiste [1], Nikolai Treiberg [1], Henry Vider [1], Siim Pikker [3], Ilona Faustova [2] and Siim Salmar [1,*]

[1] Institute of Chemistry, University of Tartu, Ravila 14A, 50411 Tartu, Estonia
[2] Institute of Bioengineering, University of Tartu, Nooruse 1, 50411 Tartu, Estonia
[3] Institute of Physics, University of Tartu, W. Ostwaldi 1, 50411 Tartu, Estonia
* Correspondence: siim.salmar@ut.ee

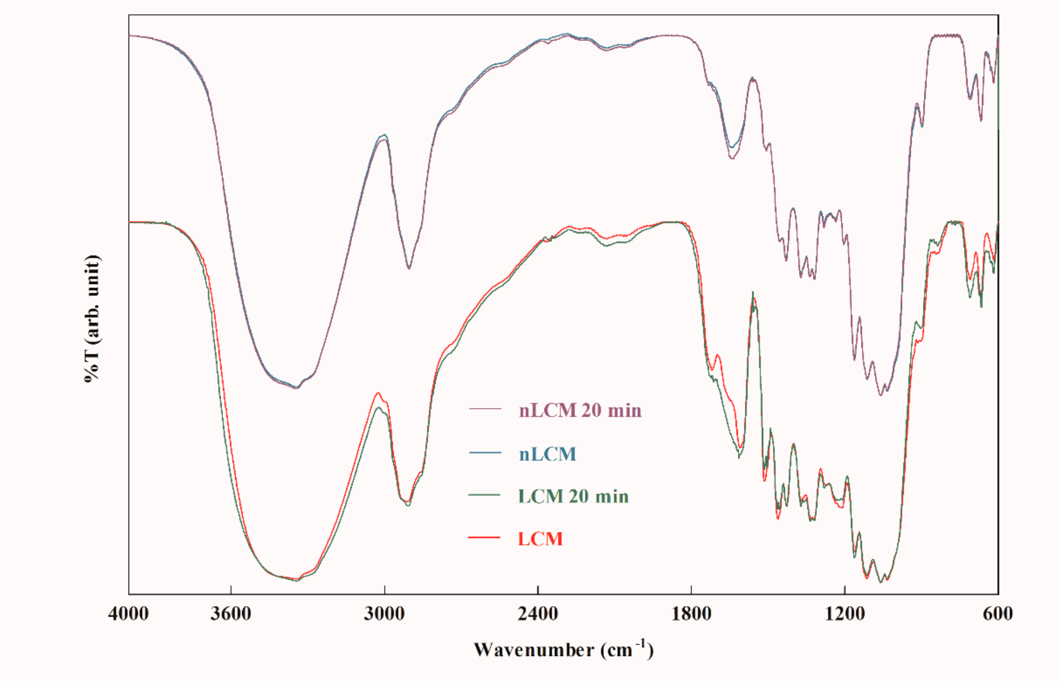

Figure S1. IR spectra for LCM and nLCM without and after 20 minutes of sonication.

Table S1. Water retention values (WRV) with standard errors for LCM and solvent treated LCMs without and after 5, 10, 20 minutes of sonication.

	**WRV ($g_{water}/g_{cellulose}$)**			
US time (min)	LCM	eLCM	tLCM	nLCM
0	3.0 ± 0.2	4.2 ± 0.3	4.4 ± 0.5	6.4 ± 0.4
5	9.2 ± 0.7	11.8 ± 1.4	17.7 ± 0.8	31.7 ± 4.9
10	8.5 ± 0.3	19.6 ± 0.9	25.5 ± 0.6	43.9 ± 8.7
20	10.2 ± 0.3	23.3 ± 2.3	31.4 ± 1.1	59.7 ± 6.1

Table S2. Percentiles of number-weighed particle size distribution for nLCM without and after 5, 10 and 20 minutes of sonication. Information in the table corresponds to **Fig. 6** in the main text.

US time (min)	MN[a] (μm)	d_{10} (μm)	d_{50} (μm)	d_{90} (μm)
0	3.7 ± 0.06	1.8 ± 0.01	2.8 ± 0.04	6.3 ± 0.12
5	1.8 ± 0.01	0.9 ± 0.003	1.4 ± 0.02	3.1 ± 0.02
10	1.8 ± 0.01	0.9 ± 0.002	1.4 ± 0.01	3.2 ± 0.009
20	0.9 ± 0.01	0.5 ± 0.001	0.6 ± 0.008	1.5 ± 0.02

[a]Mean diameter of number-weighed distribution

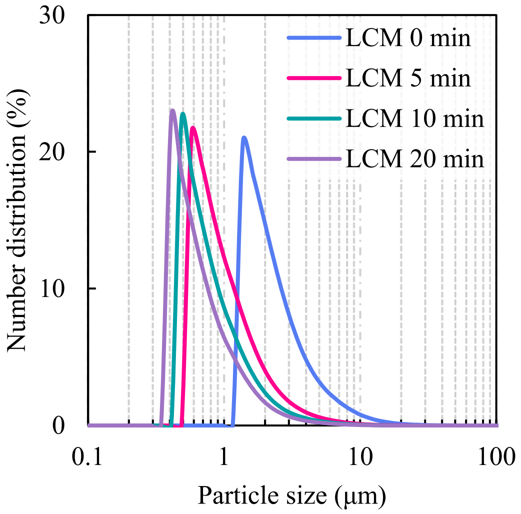

Figure S2. Number-weighed particle size distribution curves for LCM without and after 5 , 10 and 20 minutes of sonication.

Table S3. Percentiles of number-weighed particle size distribution for LCM without and after 5, 10 and 20 minutes of sonication.

US time (min)	MN[a] (µm)	d_{10} (µm)	d_{50} (µm)	d_{90} (µm)
0	2.5 ± 0.02	1.3 ± 0.01	1.8 ± 0.04	4.3 ± 0.04
5	1.3 ± 0.2	0.6 ± 0.1	0.9 ± 0.2	2.1 ± 0.3
10	0.9 ± 0.01	0.4 ± 0.001	0.6 ± 0.009	1.5 ± 0.03
20	0.7 ± 0.01	0.4 ± 0.001	0.5 ± 0.005	1.2 ± 0.01

[a]Mean diameter of number-weighed distribution

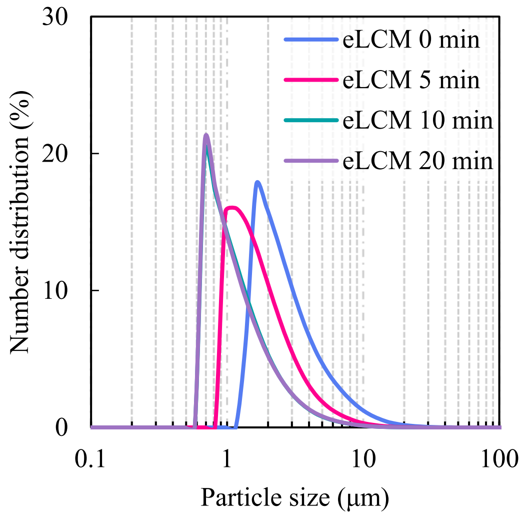

Figure S3. Number-weighed particle size distribution curves for eLCM (delignified with 70 wt% aqueous EtOH) without and after 5 , 10 and 20 minutes of sonication.

Table S4. Percentiles of number-weighed particle size distribution for eLCM without and after 5, 10 and 20 minutes of sonication.

US time (min)	MN[a] (μm)	d_{10} (μm)	d_{50} (μm)	d_{90} (μm)
0	3.0 ± 0.2	1.4 ± 0.1	2.2 ± 0.2	5.3 ± 0.4
5	1.9 ± 0.01	0.9 ± 0.004	1.4 ± 0.02	3.2 ± 0.03
10	1.3 ± 0.03	0.6 ± 0.003	0.9 ± 0.02	2.2 ± 0.06
20	1.3 ± 0.004	0.6 ± 0.003	0.9 ± 0.01	2.2 ± 0.04

[a]Mean diameter of number-weighed distribution

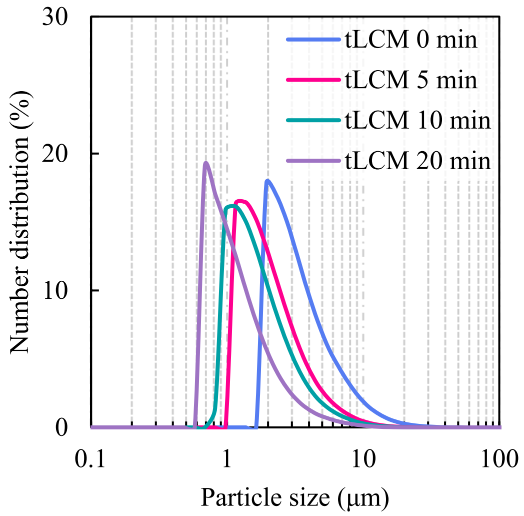

Figure S4. Number-weighed particle size distribution curves for tLCM (delignified with 70 wt% aqueous THF) without and after 5, 10 and 20 minutes of sonication.

Table S5. Percentiles of number-weighed particle size distribution for tLCM without and after 5, 10 and 20 minutes of sonication.

US time (min)	MN[a] (μm)	d_{10} (μm)	d_{50} (μm)	d_{90} (μm)
0	3.6 ± 0.08	1.8 ± 0.01	2.8 ± 0.05	6.4 ± 0.2
5	2.2 ± 0.04	1.1 ± 0.01	1.7 ± 0.04	3.7 ± 0.09
10	1.8 ± 0.1	0.9 ± 0.04	1.4 ± 0.07	3.1 ± 0.1
20	1.3 ± 0.01	0.6 ± 0.002	1 ± 0.01	2.2 ± 0.03

[a]Mean diameter of number-weighed distribution
